# Combined Piggyback Technique and Cavoportal Hemitransposition for Liver Transplant

**DOI:** 10.1155/2010/595289

**Published:** 2010-06-30

**Authors:** Jeffrey Campsen, Igal Kam

**Affiliations:** Division of Transplant Surgery, Department of Transplant, University of Colorado Denver, 1635 Aurora Ct, C318, Aurora, CO 80045, USA

## Abstract

Portal Vein thrombosis (PVT) increases the difficulty of liver transplant; however, it is not an absolute contraindication. Cavoportal hemitransposition (CPH) is an option for patients with complete PVT and no alternative collateral vein. Our center often performs the piggyback technique for the hepatic vein reconstruction, which allows for great access to the recipient vena cava in patients with known complete PVT that may need a CPH preformed to successfully restore flow to the portal system of the donor liver. We describe the use of the piggy-back technique to prepare the vena cava for possible CPH in patients with known complete PVT.

## 1. Clinical Case

A 40-year-old male with primary sclerosing cholangitis and a previous history of colectomy with j-pouch for ulcerative colitis was listed for cadaveric liver transplant with a MELD of 33. During his preoperative work-up he was found to have complete PVT. He did not appear to have any enlarged venous collateral that could be used to reconstruct the portal system during transplant. Therefore, our team was prepared to perform a CPH if needed [1, 2]. 

The patient was taken to surgery after a 16-year-old cadaveric liver was procured for him. The hepatectomy was difficult secondary to adhesions from both his inflamed liver, percutaneous biliary tubes, as well as his prior surgeries. The dissection was carried out with standard piggyback technique dissecting the liver off the vena cava to the level of the hepatic veins [3]. Thus, the vena cava was easily accessible distally to the level of the renal veins. No portal vein was found, and attempts to find suitable venous collaterals were unsuccessful.

**Figure 1:**
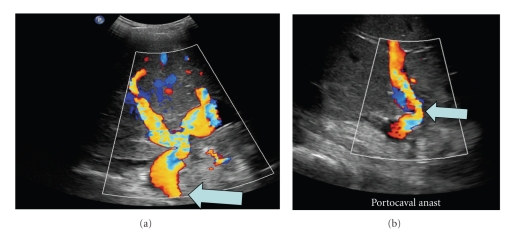
Ultrasonography documenting excellent flow through the cavoportal hemitransposition anastomosis (see arrow).

At this point there was no other option to reconstruct the portal system except to perform a CPH. Because we had prepared the vena cava of the recipient during the hepatectomy, it was easily ligated below the hepatic vein—supracaval anastomosis allowing for maximal caval length. The donor portal vein was then anastomosed to the suprarenal vena cava in an end-to-end fashion (Figure 1). Reperfusion proceeded without complication. Arterial flow was then re-established, and the biliary system was reconstructed with a choledochoduodenostomy [4].

**Figure 2:**
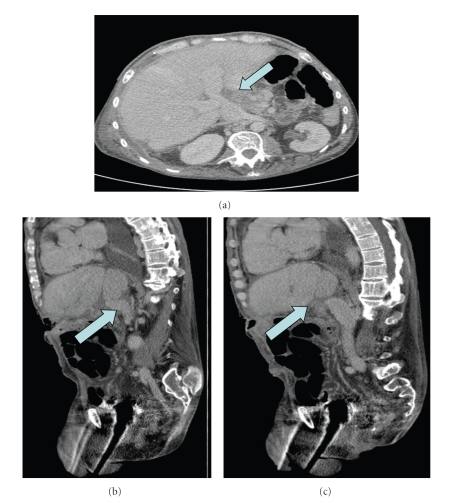
CT venogram showing cavoportal hemitransposition with the donor liver anastomosed to the recipient vena in the piggyback fashion (see arrow).

The patient had an uncomplicated postoperative course. His liver function normalized within the first week, and he has never had rejection with a 12 month followup. He did not experience any ascites, peripheral extremity edema, or renal insufficiency. Furthermore, he had return of bowel function similar to his preoperative j-pouch function. Both a postoperative ultrasonography and CT-venogram documented excellent flow through the portal vein (Figures 1 and 2).

## 2. Discussion

Portal Vein thrombosis (PVT) increases the difficulty of liver transplant; however, it is not an absolute contraindication. In patients with known preoperative complete PVT where thromboendovenectomy is impossible, alternatives for portal vein reconstruction must be preformed [5]. Cavoportal hemitransposition (CPH) is an option for patients with complete PVT and no alternative collateral veins. This technique has been previously described in [6].

Our center routinely performs the piggyback technique for the hepatic vein reconstruction. In this patient we planned to perform a vena caval preserving hepatectomy to allow for greater access to the recipient vena cava. This made the decision and the operation less difficult because the dissection of the vena cava was already completed and allowed for adequate length to reach the portal vein without using a venous jump graft. In patients with known complete PVT that may need a CPH preformed to successfully restore flow to the portal system of the donor liver we recommend preparing the patient by performing the piggyback dissection.

In conclusion, we have described the successful use of CPH combined with the piggyback technique to restore portal vein flow to the donor liver in the face of known complete PVT. The use of the piggyback technique prepares the vena cava before it is cross-clamped and allows for maximizing its length so that an end-to-end anastomosis can be preformed without a jump graft.
